# Supplementary material for: Immune-Mediated Effects of Microplanar Radiotherapy with a Small Animal Irradiator
Source: Cancers (Basel). 2021 Dec 29;14(1):155. doi: 10.3390/cancers14010155 (PMC8750301; doi:10.3390/cancers14010155)
Supplement: Supplementary file 1 [file cancers-14-00155-s001.zip › cancers-1438544-supplementary.pdf]

cancers

MDPI

Supplementary

Method S1: Immunohistochemistry Staining, Image acquisition and analysis

Immunohistochemical analysis for CD45R/B220 (550286, BD Pharmingen) was performed on paraffin slide specimens. Antigen retrieval was performed using Ventana's CC1 (pH 8.5), for 40 mins at 100 °C, given a protein block for 1 hour, followed by a peroxidase incubation for 12 mins. Next, the primary antibody diluent (1:500) was added for 1 hr at room temperature using Discovery PSS Diluent, 760-212, followed by the secondary antibody (Ventana Omap OmniMap anti-Rat HRP, 760-4457, Ready to Use) for 32 minutes at room temperature. The slides were incubated in Discovery Purple, 760-229 for 20 mins.

Immunohistochemical analysis for Mouse CD4 (14-9766, eBioscience) was performed on paraffin slide specimens. Antigen retrieval was performed using Ventana's CC1 (pH 8.5), for 72 mins at 100 °C, followed by the primary antibody diluent (1:25) for 4 hrs at room temperature using Discovery Ab Diluent, 760-108. The slides were then given a post primary peroxidase incubation for 8 mins, followed by the secondary antibody (Ventana Omap OmniMap anti-Rat HRP, 760-4457, Ready to Use) for 32 minutes at room temperature. The slides were incubated in Discovery Purple, 760-229 for 1 hr and 32 minutes.

Immunohistochemical analysis for anti-Mouse CD8a (14-0808, eBioscience) was performed on paraffin slide specimens. Antigen retrieval was performed using Ventana's CC1 (pH 8.5), for 64 minutes at 100° C and given a peroxidase step for 8 mins, followed by the primary antibody diluent (1:100) for 2 hours at room temperature using Discovery PSS Diluent, 760-212, and then the secondary antibody (Ventana Omap OmniMap anti Rat HRP, 760-4457, Ready to Use) for 32 minutes at room temperature. The slides were incubated in Discovery Purple, 760-229 for 32 mins.

Immunohistochemical analysis for Rat Anti-Mouse F4/80 Antibody: CI:A3-1 (AbD Serotec, Cat # MCA497RT) was performed on paraffin slide specimens. Antigen retrieval was performed with Protease 2 (760-2019) for 8 mins at 37 °C and given a protein block for 32 mins. Next, the slides were incubated in the primary antibody diluent (1:25) for 1 hr at 42° C using AB Discovery Diluent (760-108), followed by the secondary antibody (Ventana Omap OmniMap anti Rat HRP, 760-4457, Ready to Use) for 32 minutes at room temperature, and a post peroxidase step for 12 minutes. The slides were incubated in Discovery Purple, 760-229 for 32 mins.

Immunohistochemical analysis for FoxP3 (14-5773, eBioscience) was performed on paraffin slide specimens. Antigen retrieval was performed using Ventana's CC1 (pH 8.5), for 64 minutes at 100 °C, given a protein block for 1 hr, followed by a peroxidase incubation for 8 minutes. The primary antibody diluent (1:25) was added and incubated for 2 hrs at room temperature using Discovery Ab Diluent, 760-108. The slides were then given the secondary antibody (Ventana Omap OmniMap anti-Rat HRP, 760-4457, Ready to Use) for 32 minutes at room temperature. The slides were incubated in Discovery Purple, 760-229 for 1 hr and 4 minutes.

All the slides were counterstained with Hematoxylin II for 12 minutes, and then Bluing Reagent for 4 mins. The slide staining was performed using Ventana's Discovery Ultra Automated IHC staining system.

Table S1. RT-qPCR primer List.

Gene Name	Forward	Reverse
B-actin	GGCTGTATTCCCCTCCATCG	CCAGTTGG-TAACAATGCCATGT
CCL5	GCTGCTTTGCCTACCTCTCC	TCGAGTGACAAACAC-GACTGC
IFNB1	CAGCTCCAAGAAAGGAC-GAAC	GGCAGTGTAACTCTTCTG-CAT
CXCL9	GGAGTTCGAG-GAACCCTAGTG	GGGATTTGTAGTG-GATCGTGC
PD-L1	GCTCCAAAGGACTTGTAC-GTG	TGATCTGAAGGGCAG-CATTTC
